# A dataset of exogenous variables and historical electricity demand for short-term load forecasting of the national interconnected electric system (SENI) in the Dominican Republic from 2021 to 2024

**DOI:** 10.1016/j.dib.2025.112057

**Published:** 2025-09-16

**Authors:** Rafael Orlando Uceta-Acosta, Deyslen Mariano-Hernandez, Yeulis Rivas-Peña, Víctor S. Ocaña-Guevara, Miguel Aybar-Mejía, Máximo A. Domínguez-Garabitos

**Affiliations:** a Área de Ciencias Básicas, Instituto Tecnológico de Santo Domingo, Santo Domingo 10602, Dominican Republic; b Área de Ingeniería, Instituto Tecnológico de Santo Domingo, Santo Domingo 10602, Dominican Republic; c Facultad de Ingeniería Mecánica e Industrial, Universidad Central “Marta Abreu” de Las Villas (UCLV), Santa Clara, Cuba

**Keywords:** Electricity demand forecasting, Short-term forecasting, Exogenous variables, Time series, Energy modelling, Machine learning

## Abstract

This dataset contains historical records of electricity demand in the Dominican Republic from January 2021 to December 2024, with hourly resolution. It was compiled to support short-term load forecasting of the National Interconnected Electric System (SENI). The dataset includes the total system demand in megawatts (MW), along with a set of exogenous variables commonly used in forecasting models. These variables include weather data retrieved from Open-Meteo (such as temperature and humidity), time-lagged demand features, and calendar-based indicators (e.g., weekends, holidays, month, hour). All data were collected from open sources, including the official website of the electricity market and system operator, the Organismo Coordinador (OC), as well as public meteorological APIs.

The dataset is structured and cleaned to be directly usable for time series modeling applications. It can be reused by researchers, utility planners, and data scientists for benchmarking forecasting models, developing predictive tools, or supporting energy planning tasks in tropical, developing power systems. The data is provided in CSV format.

**Nomenclature**
SENI: National Interconnected Electric System
MW: Megawatts
ML: Machine Learning
CSV: Comma-Separated Values
OC: Organismo Coordinador
IQR: Inter Quartile Range

Specifications Table

**Table:** 

Subject	Energy	
Specific subject area	Short-term electricity demand forecasting with exogenous and lagged variables.	
Data format	CSV file	
Type of data	Table, Chart, Processed	
Data collection	Electricity demand data from 2021 to 2024 were collected from the Dominican Republic’s system operator as monthly Excel files with hourly resolution and inconsistent structure, then manually unified using Excel. Only active power (MW) was retained, while apparent and reactive power were discarded. Outliers were validated against OC reports and kept. Weather data were retrieved from Open-Meteo per province and averaged. Calendar features and demand lags (1–24 h) were engineered. Low-correlation variables were excluded. Data were aligned at hourly frequency without normalization.	
Data source location	Below is a list of primary data sources.	
	Primary data sources	References
	1. Organismo Coordinador del Sistema Eléctrico Nacional Interconectado (OC)	[1]
	2. Open Meteo	[2]
Data accessibility	Repository: Kaggle - Short-Term Forecasting Dataset SENI Dominican Rep URL: https://www.kaggle.com/datasets/rafaeluceta/short-term-forecasting-dataset-seni-dominican-rep License: CC BY-NC-SA 4.0	

## 1 Value of the Data

• This dataset is valuable for researchers, data scientists, and energy planners interested in short-term electricity demand forecasting using machine learning and statistical models.
• The dataset enables the analysis of the impact of exogenous variables (weather and calendar data) and lagged demand variables on electricity consumption patterns in the Dominican Republic.
• Data can be reused to develop, test, and compare forecasting models, thereby improving operational planning, grid stability, and energy efficiency in the Dominican Republic and other Small Island Developing States with similar energy systems.
• By providing unified, pre-processed, and ready-to-use hourly data, the dataset helps overcome barriers related to data inaccessibility and heterogeneity from raw data sources.
• It can also be used in educational and methodological settings to teach time series forecasting, feature engineering, and benchmarking of machine learning models, with potential applicability in other domains where exogenous time-series data are common (e.g., transportation, retail demand, healthcare).
• This dataset can complement previous studies on electricity demand forecasting in developing countries [[1], [2], [3], [4]].

## 2 Objective

Provide a structured, unified, and open-access hourly dataset of electricity demand along with exogenous and lagged variables to support the development of short-term forecasting models for the Dominican Republic's SENI. The dataset aims to facilitate data-driven decision-making, improve operational planning, and enable energy analysts and researchers to explore the impact of weather conditions, calendar effects, and historical consumption patterns on electricity demand. By making this data publicly available, the dataset contributes to advancing the use of machine learning and statistical modelling for electricity demand forecasting in Small Island Developing States and similar contexts. This is the first curated open-access dataset of its kind for the Dominican Republic and the wider Caribbean region, filling a critical gap in publicly available electricity demand data.

## 3 Data Description

The dataset is provided as a single CSV file named “datos_modelo.csv”, containing hourly records from January 1, 2021, to December 31, 2024. Data prior to 2021 were excluded due to concerns about reliability and the structural disruptions in electricity demand caused by the COVID-19 pandemic, which significantly altered consumption patterns. The Organismo Coordinador (OC) provided the dataset upon request for “all data available from 2021 to the present,” with validated records available only up to 2024. Each row in the file represents one hour of data, while each column corresponds to one of the 32 variables used for short-term electricity demand forecasting. These variables are grouped into three main categories: electricity demand, meteorological variables, and calendar features. Table 1 provides an overview of the variables included, and Illustration 1 shows a data snippet with the top 5 records Table 2.

**Table 1:** Dataset variables in CSV file.

Variable’s Group	Name	Name in Dataset	Unit
Electricity Demand	Active Power	active_power_mw	MW
Electricity Demand	Active Power Lags 1	active_power_lag_1h	MW
Electricity Demand	Active Power Lags 2	active_power_lag_2h	MW
Electricity Demand	Active Power Lags 3	active_power_lag_3h	MW
Electricity Demand	Active Power Lags 4	active_power_lag_4h	MW
Electricity Demand	Active Power Lags 5	active_power_lag_5h	MW
Electricity Demand	Active Power Lags 6	active_power_lag_6h	MW
Electricity Demand	Active Power Lags 7	active_power_lag_7h	MW
Electricity Demand	Active Power Lags 8	active_power_lag_8h	MW
Electricity Demand	Active Power Lags 9	active_power_lag_9h	MW
Electricity Demand	Active Power Lags 10	active_power_lag_10h	MW
Electricity Demand	Active Power Lags 11	active_power_lag_11h	MW
Electricity Demand	Active Power Lags 12	active_power_lag_12h	MW
Electricity Demand	Active Power Lags 13	active_power_lag_13h	MW
Electricity Demand	Active Power Lags 14	active_power_lag_14h	MW
Electricity Demand	Active Power Lags 15	active_power_lag_15h	MW
Electricity Demand	Active Power Lags 16	active_power_lag_16h	MW
Electricity Demand	Active Power Lags 17	active_power_lag_17h	MW
Electricity Demand	Active Power Lags 18	active_power_lag_18h	MW
Electricity Demand	Active Power Lags 19	active_power_lag_19h	MW
Electricity Demand	Active Power Lags 20	active_power_lag_20h	MW
Electricity Demand	Active Power Lags 21	active_power_lag_21h	MW
Electricity Demand	Active Power Lags 22	active_power_lag_22h	MW
Electricity Demand	Active Power Lags 23	active_power_lag_23h	MW
Electricity Demand	Active Power Lags 24	active_power_lag_24h	MW
Meteorological	Cloud Cover	cloud_cover_percent	%
Meteorological	Apparent Temperature	apparent_temperature_c	Celsius
Calendar	Month	month	-
Calendar	Hour	hour	-
Calendar	Holiday	is_holiday	0/1
Calendar	Weekend	is_weekend	0/1
Calendar	School Vacation	is_school_vacation	0/1

**Illustration 1:**
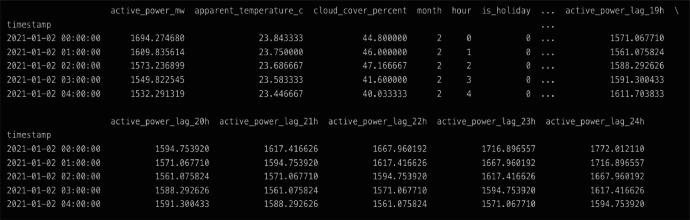
Data snippet of the CSV file with the top 5 rows.

**Table 2:** Geographic coordinates used to retrieve hourly weather data for each province in the Dominican Republic.

Province	City	Latitude	Longitude
Distrito Nacional	Santo Domingo	18.4861	−69.9312
Azua	Azua de Compostela	18.4539	−70.7346
Bahoruco	Neiba	18.481	−71.419
Barahona	Barahona	18.2085	−71.1008
Dajabón	Dajabón	19.5482	−71.7083
Duarte	San Francisco de Macorís	19.3006	−70.2536
El Seibo	El Seibo	18.765	−69.0381
Espaillat	Moca	19.3933	−70.5252
Hato Mayor	Hato Mayor del Rey	18.7664	−69.2567
Hermanas Mirabal	Salcedo	19.3773	−70.4179
Independencia	Jimaní	18.4916	−71.849
La Altagracia	Higüey	18.615	−68.707
La Romana	La Romana	18.4273	−68.9728
La Vega	La Vega	19.222	−70.5293
María Trinidad Sánchez	Nagua	19.3834	−69.8476
Monte Cristi	San Fernando de Monte Cristi	19.8486	−71.645
Monte Plata	Monte Plata	18.807	−69.7843
Pedernales	Pedernales	18.0381	−71.7411
Peravia	Baní	18.2796	−70.3318
Puerto Plata	Puerto Plata	19.7965	−70.6884
Samaná	Santa Bárbara de Samaná	19.205	−69.3369
San Cristóbal	San Cristóbal	18.4154	−70.1051
San José de Ocoa	San José de Ocoa	18.5463	−70.5069
San Juan	San Juan de la Maguana	18.8059	−71.229
San Pedro de Macorís	San Pedro de Macorís	18.45	−69.3
Sánchez Ramírez	Cotuí	19.05	−70.15
Santiago	Santiago de los Caballeros	19.45	−70.7
Santiago Rodríguez	San Ignacio de Sabaneta	19.4683	−71.34
Santo Domingo	Santo Domingo Este	18.4885	−69.857
Valverde	Mao	19.5519	−71.077
Elías Piña	Comendador	18.8708	−71.7075
Monseñor Nouel	Bonao	18.9428	−70.4084

Recent advances in short-term electricity demand forecasting increasingly emphasize hybrid and deep learning architectures that leverage exogenous variables to enhance accuracy [5]. For instance, Ugbehe [6] provides a comprehensive review that highlights the growing dominance of hybrid methodologies, which outperform traditional statistical models. Demir [7] demonstrates that customized LSTM/CNN ensembles incorporating temperature, humidity, and wind features yield significantly improved forecasts. Similarly, Chen [8] deploys an enhanced Inception-V4 model optimized with weather-related inputs to outperform conventional forecasting techniques. These advancements underscore the timely relevance of providing a robust, pre-processed dataset with exogenous features, as presented in this article.

### 3.1 Electricity demand and lags

The variable “active_power_mw” represents the total hourly electricity demand of the Dominican Republic’s SENI, measured in megawatts (MW). It was obtained directly from the records available on the OC website and serves as the target variable for forecasting applications.

To capture short-term temporal dependencies in demand behavior, 24 lagged variables were generated, from active_power_lag_1 to active_power_lag_24. Each of these variables corresponds to the value of active_power_mw observed 1 to 24 h prior, respectively. These lagged features are commonly used in time series models to enhance predictive performance and allow models to learn from recent demand history.

**Fig. 1:**
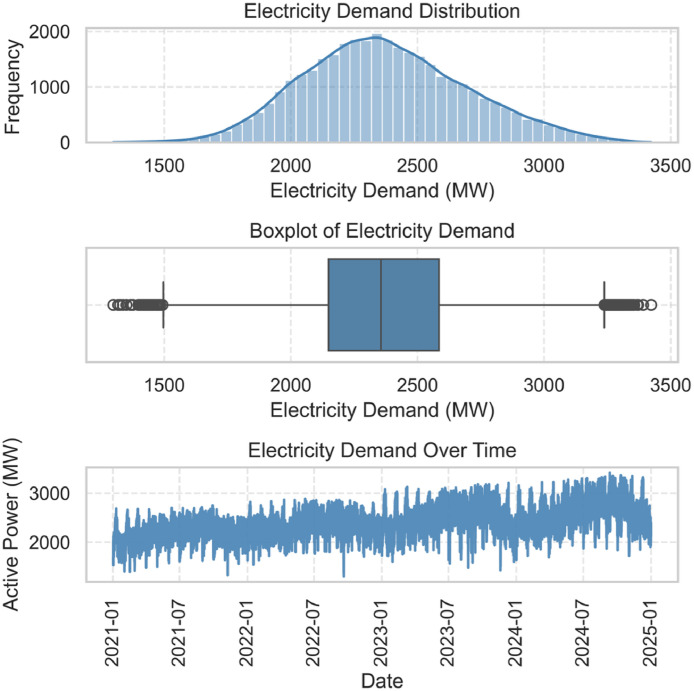
Distribution, boxplot, and time series of hourly electricity demand (active_power_mw).

A summary of the active_power_mw variable is presented in Fig. 1. The top panel displays a histogram with kernel density estimation, showing the distribution of hourly electricity demand. The middle panel presents a boxplot of the same variable, allowing a visual inspection of its dispersion and extreme values. The bottom panel shows the time series evolution of active_power_mw from 2021 to 2024 with no missing values.

### 3.2 Meteorological variables

This group includes weather-related variables retrieved from the Open-Meteo API [9]. For each of the 32 provinces in the Dominican Republic, one representative coordinate was selected, and hourly weather data were collected for the entire study period (2021–2024). The national values were obtained by averaging the corresponding variable across all provinces at each timestamp.

**Fig. 2:**
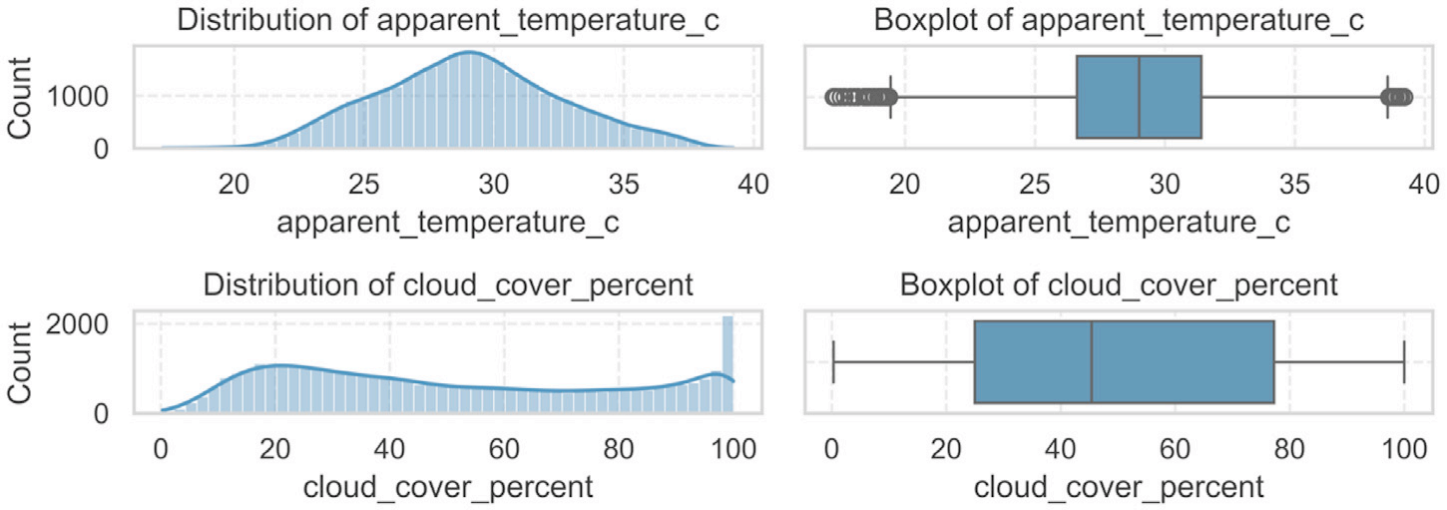
Distribution and boxplots of the meteorological variables: Apparent Figure2: Temperature (apparent_temperature_c) and Cloud Cover (cloud_cover_percent).

Each variable is available at hourly resolution and aligned with the electricity demand time series. Variables such as rainfall, humidity, and wind speed were initially retrieved but excluded from the final dataset because of their low correlation with the target variable. Fig. 2 provides a visual summary of the meteorological variables included in the dataset. The top row shows the distribution and boxplot of apparent_temperature_c (Apparent Temperature) [1,3], while the bottom row displays the same for cloud_cover_percent (Cloud Cover). These visualizations help to illustrate the range, central tendency, and dispersion of each variable.

### 3.3 Calendar variables

This group includes time-based categorical variables manually derived from the timestamp of each observation. All variables in this group are encoded at an hourly resolution and aligned with the electricity demand time series [1]. The calendar variables are:

• month: Month of the year (1–12).
• hour: Hour of the day (0–23).
• is_holiday: Binary indicator (1 if the date is a national holiday, 0 otherwise). National holidays were identified using the Python holidays library, which relies on the official Dominican Republic public holiday calendar.
• is_weekend: Binary indicator (1 if the date falls on a weekend, 0 if a weekday).
• is_school_vacation: Binary indicator (1 if the date falls within official school vacation periods, 0 otherwise). School vacation periods were defined based on the academic calendars published annually by the Ministry of Education of the Dominican Republic.

These variables were engineered to incorporate periodic and event-driven effects into the forecasting models. All calendar variables are included in the same CSV file as the other predictors and span the whole time range of the dataset (January 2021 to December 2024), with no missing values.

## 4 Experimental Design, Materials and Methods

This section details the procedures followed to construct and pre-process the dataset used for short-term electricity demand forecasting. The data pipeline was designed to integrate information from multiple sources and transform it into a single, clean, and analysis-ready dataset with hourly resolution. Variables were grouped into three categories: electricity demand, meteorological variables, and calendar variables. Each group underwent specific pre-processing steps, which are described in the following subsections. All transformations, exclusions, and imputations were implemented using Python and open-source libraries.

### 4.1 Electricity demand variables

Hourly electricity demand data for the Dominican Republic were requested from the system operator (Organismo Coordinador) and provided in monthly Excel files covering the period from January 2021 to December 2024. According to [10], the system operator is responsible for scheduling and forecasting electricity demand. Data prior to 2021 were excluded due to concerns about reliability and the structural disruptions in electricity demand caused by the COVID-19 pandemic, which significantly altered standard consumption patterns. At the time of dataset construction, validated records were available up to December 2024, which were consolidated to provide the most recent data possible. These files, initially delivered with inconsistent structures, were manually standardized and merged into a single, long-format time series using Excel. Only active power (active_power_mw, in megawatts) was retained; reactive and apparent power measurements were discarded.

**Fig. 3:**
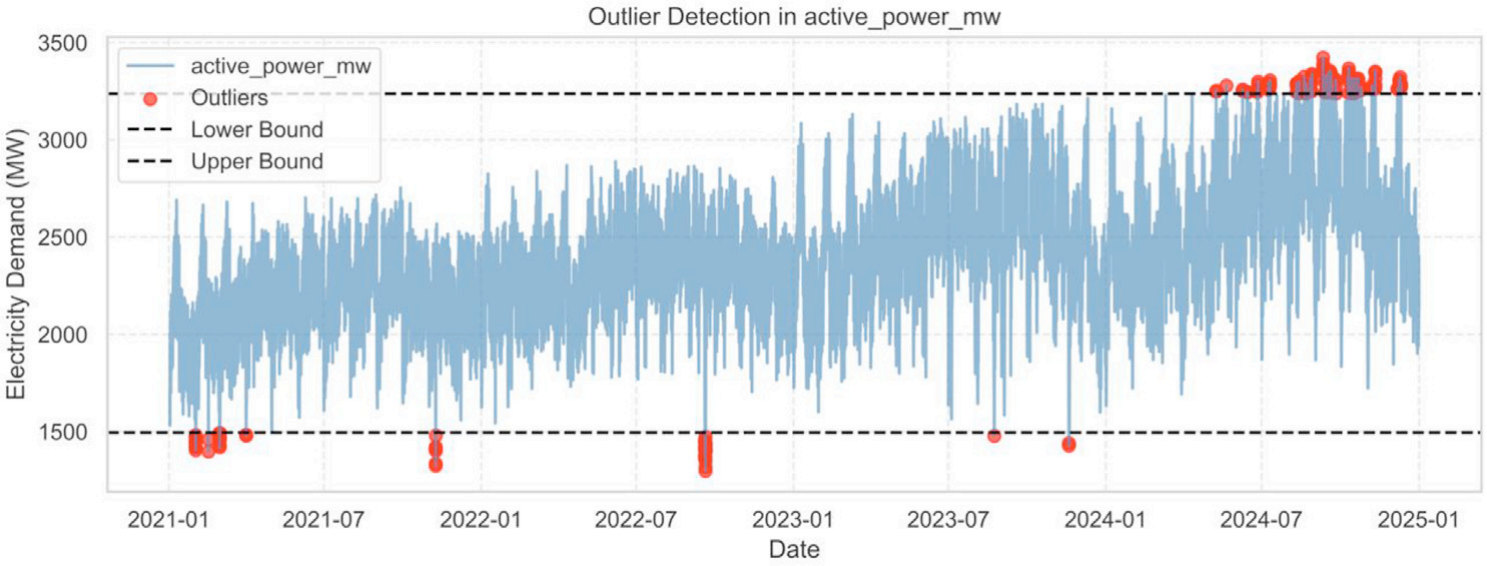
Outliers in the electricity demand variable (active_power_mw).

Outlier detection was performed using interquartile range (IQR) analysis and visualized through boxplots in Fig. 1 and line plots in Fig. 3. Outliers were not removed, as they corresponded to real events such as holidays or system incidents, as verified with the OC website’s dashboards.

**Fig. 4:**
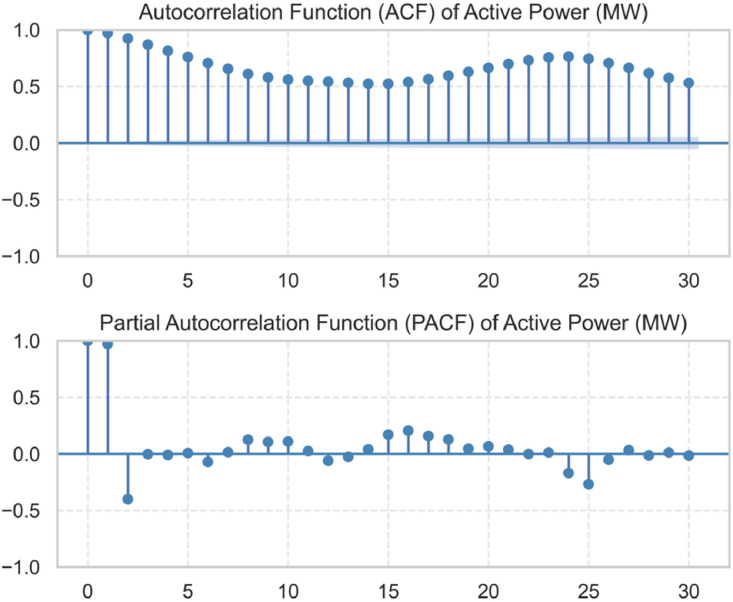
Autocorrelation function (ACF) and Partial Autocorrelation Function (PACF) of the electricity demand variable (active_power_mw) over the first 30 hourly lags.

To assess the temporal autocorrelation structure of the demand signal, both the Autocorrelation Function (ACF) and the Partial Autocorrelation Function (PACF) were computed using the statsmodels Python library. As shown in Fig. 4, the ACF reveals strong positive autocorrelation within the first 24 hourly lags, indicating a high degree of persistence in the demand signal. The PACF further confirms that the first few lags (especially lag 1 and lag 2) have significant partial correlations with the current demand value, even after controlling for intermediate values. Based on these observations, 24 hourly lag features were included in the dataset, using the “shift” function in Pandas, to capture short-term temporal. Dependencies relevant for forecasting models.

### 4.2 Meteorological variables

Hourly meteorological data were collected from the Open-Meteo API (https://open-meteo.com/), an open-source weather data service. For each of the 32 provinces of the Dominican Republic, one representative geographic coordinate was selected. The weather data was downloaded in JSON format using automated Python scripts and converted to Pandas DataFrames.

The following variables were initially retrieved: air temperature, apparent temperature, humidity, rainfall, cloud cover, and wind speed. All variables were recorded at hourly resolution and then averaged across provinces to obtain a national-level indicator for each timestamp.

After a correlation analysis shown in Fig. 6 with the target variable (active_power_mw), only two meteorological variables were retained in the final dataset due to their stronger association with electricity demand:

• apparent_temperature_c (Apparent Temperature), in degrees Celsius.
• cloud_cover_percent (Cloud Cover), in percentage.

**Fig. 5:**
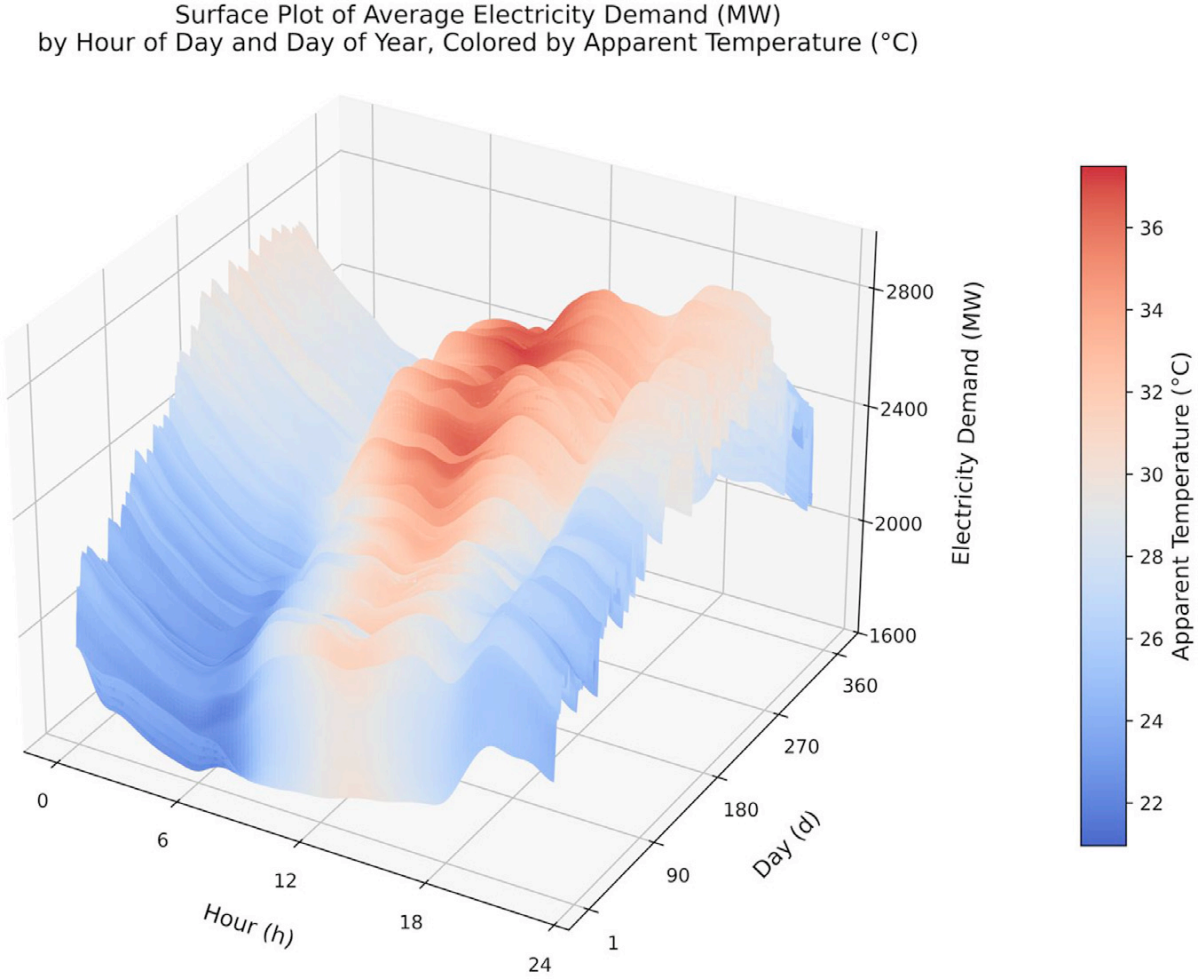
Interpolated 3D surface of hourly average electricity demand (active_power_mw) by hour of day and day of year. Surface color represents apparent temperature (apparent_temperature_c) in °C.

To explore the interaction between temperature and electricity demand, a 3D surface plot was generated with the hour of the day on the x-axis, the day of the year on the y-axis, and the electricity demand (active_power_mw) on the z-axis. Apparent temperature (apparent_temperature_c) was used to color the surface, interpolated to enhance visual continuity. As shown in Fig. 5, higher apparent temperatures tend to coincide with elevated demand, particularly during midday and summer months.

All meteorological variables are stored in the same CSV file as the rest of the dataset and are fully aligned with the hourly timestamps of the demand data.

### 4.3 Calendar variables

Calendar-based features were generated from the hourly timestamp using the pandas, datetime, and holidays libraries in Python. These features were designed to capture temporal patterns in electricity demand related to the time of day, calendar structure, and national events. All calendar variables are aligned to the hourly resolution of the dataset and stored in the same CSV file as the other predictors.

The following variables were created:

• month: Month of the year (1–12).
• hour: Hour of the day (0–23).
• is_holiday: Binary flag set to 1 if the date corresponds to an official Dominican Republic holiday, using the holidays package.
• is_weekend: Binary flag indicating whether the date is a weekend (1 for Saturday or Sunday, 0 otherwise).
• is_school_vacation: Binary flag indicating whether the hour falls within official school vacation periods.

School vacation periods were defined manually using known academic break dates from 2021 to 2024, including summer and end-of-year holidays. Each period was encoded by assigning a value of 1 to the corresponding timestamps.

All calendar features are numerically encoded to ensure compatibility with forecasting models. Missing values are not present, as all variables were deterministically generated from the complete date range of the dataset.

### 4.4 Variable selection process

**Fig. 6:**
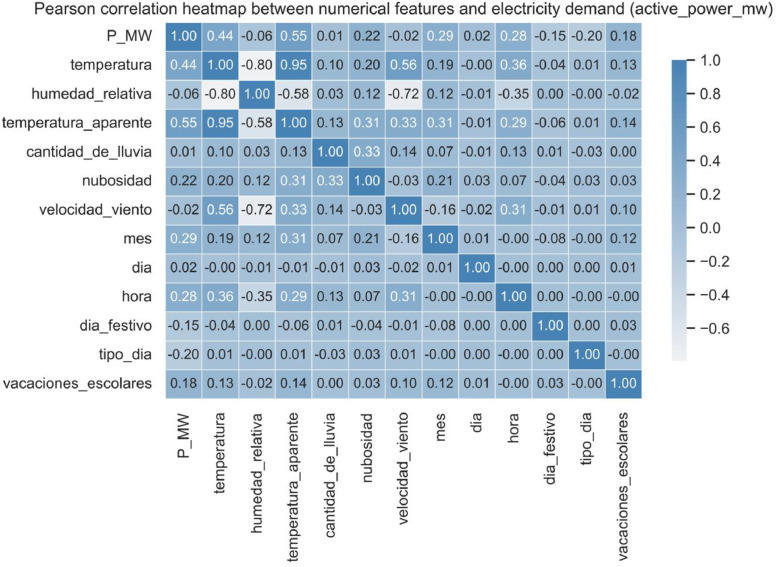
Pearson correlation heatmap between numerical features and electricity demand (active_power_mw).

After constructing the complete set of predictor variables, a correlation analysis was conducted to evaluate their linear relationship with the target variable (active_power_mw). Pearson’s correlation coefficient was calculated for all numerical predictors using the “corr” method from the Pandas library. The resulting heatmap is shown in Fig. 6.

As a selection criterion, only variables with an absolute correlation coefficient greater than 0.1 with the target were considered for inclusion. In addition, when two variables were found to be strongly correlated with each other (e.g., temperature_c and apparent_temperature_c), only the one with stronger correlation to the target was retained to avoid redundancy.

Specifically, precipitation_mm, relative_humidity_percent, and wind_speed_kph were excluded because of their weak correlation with the target variable. The variable day (day of the month) was also discarded due to its lack of predictive value. Furthermore, temperature_c was removed because it exhibited high correlation with apparent_temperature_c, which was kept due to its slightly stronger association with electricity demand.

The final dataset includes features that either demonstrated a meaningful statistical relationship with the target or are widely recognized in the literature for short-term load forecasting, such as hour, month, calendar indicators (holiday, weekend, school vacation), and the first 24 hourly lags of active power. This selection strikes a balance between dimensionality reduction and model interpretability.

## Limitations

Although the dataset was carefully assembled from open-access and official sources, certain limitations are inherent in the data collection process. First, the electricity demand data obtained from the system operator was distributed across monthly Excel files with inconsistent formatting, requiring manual validation and increasing the risk of structural inconsistencies. Second, weather data were collected from only one geographic location per province and then averaged to represent national conditions. This spatial simplification may not fully reflect localized climate variations that impact electricity demand in specific areas. Third, historical demand data prior to 2021 were not included due to structural disruptions caused by the COVID-19 pandemic. Similarly, the dataset extends only until December 2024, as this was the latest point for which complete and validated records were received. Finally, some variables, such as school vacation periods and national holidays, were manually encoded based on publicly available calendars. These assignments may not accurately capture local variations or unannounced changes in the academic or public calendar.

## Ethics statements

The authors declare that they did not conduct human or animal studies. The authors declare that they did not collect social media data and did not need permission to use the primary data.

## CRediT Author Statement

**Rafael Orlando Uceta**-**Acosta, Yeulis Rivas**-**Peña, Víctor S. Ocaña-Guevara, Miguel Aybar-Mejía, Máximo A. Domínguez-Garabitos**: Conceptualization, **Rafael Orlando Uceta**-**Acosta, Víctor S. Ocaña-Guevara, Máximo A. Domínguez-Garabitos**: methodology, **Rafael Orlando Uceta**-**Acosta, Yeulis Rivas**-**Peña, Víctor S. Ocaña-Guevara, Máximo A. Domínguez-Garabitos**: writing—original draft preparation, **Rafael Orlando Uceta**-**Acosta, Deyslen Mariano-**-**Hernandez, Yeulis Rivas**-**Peña, Víctor S. Ocaña-Guevara, Miguel Aybar-Mejía, Máximo A. Domínguez-Garabitos**: writing—review and editing, **Deyslen Mariano-**-**Hernandez, Yeulis Rivas**-**Peña, Víctor S. Ocaña-Guevara, Miguel Aybar-Mejía, Máximo A. Domínguez-Garabitos**: supervision. All authors have read and agreed to the published version of the manuscript.

## Figures and Tables

**Table 3:** Holidays and school vacation details.

Category	Date Range	Description
National Holiday	2021–01–01	New Year's Day
National Holiday	2021–01–21	Our Lady of Altagracia
National Holiday	2021–02–27	Independence Day
National Holiday	2021–08–16	Restoration Day
National Holiday	2021–09–24	Our Lady of Mercedes
National Holiday	2021–12–25	Christmas Day
National Holiday	2022–01–01	New Year's Day
National Holiday	2022–01–21	Our Lady of Altagracia
National Holiday	2022–02–27	Independence Day
National Holiday	2022–08–16	Restoration Day
National Holiday	2022–09–24	Our Lady of Mercedes
National Holiday	2022–12–25	Christmas Day
National Holiday	2023–01–01	New Year's Day
National Holiday	2023–01–21	Our Lady of Altagracia
National Holiday	2023–02–27	Independence Day
National Holiday	2023–08–16	Restoration Day
National Holiday	2023–09–24	Our Lady of Mercedes
National Holiday	2023–12–25	Christmas Day
National Holiday	2024–01–01	New Year's Day
National Holiday	2024–01–21	Our Lady of Altagracia
National Holiday	2024–02–27	Independence Day
National Holiday	2024–08–16	Restoration Day
National Holiday	2024–09–24	Our Lady of Mercedes
National Holiday	2024–12–25	Christmas Day
School Vacation	2021–12–20 to 2022–01–10	Christmas Break
School Vacation	2022–07–01 to 2022–08–31	Summer Break
School Vacation	2022–12–20 to 2023–01–10	Christmas Break
School Vacation	2023–07–01 to 2023–08–31	Summer Break
School Vacation	2023–12–20 to 2024–01–10	Christmas Break
School Vacation	2024–07–01 to 2024–08–31	Summer Break

## Data Availability

KaggleShort-Term Forecasting Dataset SENI Dominican Rep (Reference data)

## Appendix

see Table 3.
